# Treatment with anagliptin, a DPP-4 inhibitor, decreases FABP4 concentration in patients with type 2 diabetes mellitus at a high risk for cardiovascular disease who are receiving statin therapy

**DOI:** 10.1186/s12933-020-01061-0

**Published:** 2020-06-15

**Authors:** Masato Furuhashi, Ichiro Sakuma, Takeshi Morimoto, Yukimura Higashiura, Akiko Sakai, Megumi Matsumoto, Mio Sakuma, Michio Shimabukuro, Takashi Nomiyama, Osamu Arasaki, Koichi Node, Shinichiro Ueda

**Affiliations:** 1 Department of Cardiovascular, Renal and Metabolic Medicine, Sapporo Medical University School of Medicine, South 1, West 16, Sapporo, 060-8543 Japan; 2 Caress Sapporo Hokko Memorial Clinic, Sapporo, Japan; 3 Department of Clinical Epidemiology, Hyogo College of Medicine, Nishinomiya, Japan; 4 Department of Diabetes, Endocrinology and Metabolism, Fukushima Medical University, Fukushima, Japan; 5 Department of Diabetes, Metabolism and Endocrinology, International University of Health and Welfare Ichikawa Hospital, Ichikawa, Japan; 6 Department of Cardiology, Tomishiro Central Hospital, Tomigusuku, Japan; 7 Department of Cardiovascular Medicine, Saga University, Saga, Japan; 8 Department of Pharmacology and Therapeutics, University of the Ryukyus, Nishihara, Japan

**Keywords:** Anagliptin, Sitagliptin, Dipeptidyl peptidase-4 inhibitor, Fatty acid-binding protein

## Abstract

**Background:**

Fatty acid-binding protein 4 (FABP4) acts as a novel adipokine, and elevated FABP4 concentration is associated with obesity, insulin resistance and atherosclerosis. Dipeptidyl peptidase-4 (DPP-4) inhibitors, a class of antidiabetic drugs, have distinct structures among the drugs, possibly leading to a drug class effect and each drug effect. Sitagliptin, a DPP-4 inhibitor, has been reported to decrease FABP4 concentration in drug-naïve and sulfonylurea-treated patients with type 2 diabetes mellitus. Anagliptin, another DPP-4 inhibitor, was shown to decrease low-density lipoprotein cholesterol (LDL-C) level to a greater extent than that by sitagliptin in the Randomized Evaluation of Anagliptin vs. Sitagliptin On low-density lipoproteiN cholesterol in diabetes (REASON) trial.

**Aim and methods:**

As a sub-analysis study using data obtained from the REASON trial, we investigated the effects of treatment with anagliptin (n = 148, male/female: 89/59) and treatment with sitagliptin (n = 159, male/female: 93/66) for 52 weeks on FABP4 concentration in patients with type 2 diabetes mellitus at a high risk for cardiovascular events who were receiving statin therapy.

**Results:**

The DPP-4 inhibitor had been administered in 82% of the patients in the anagliptin group and 81% of the patients in sitagliptin group prior to randomization. Serum FABP4 level was significantly decreased by 7.9% by treatment with anagliptin (P = 0.049) and was not significantly decreased by treatment with sitagliptin (P = 0.660). Change in FABP4 level was independently associated with basal FABP4 level and changes in waist circumference and creatinine after adjustment of age, sex and the treatment group.

**Conclusion:**

Anagliptin decreases serum FABP4 concentration independent of change in hemoglobin A1c or LDL-C in patients with type 2 diabetes mellitus and dyslipidemia who are on statin therapy.

*Trial registration* ClinicalTrials.gov number NCT02330406. Registered January 5, 2015, https://clinicaltrials.gov/ct2/show/NCT02330406

## Background

Fatty acid-binding proteins (FABPs) are about 14–15-kDa predominantly cytosolic proteins that can reversibly bind long-chain fatty acids with high affinity and facilitate the transport of lipids to specific compartments in the cell [1, 2]. Among FABPs, fatty acid binding protein 4 (FABP4), also referred to as adipocyte FABP or aP2, is mainly expressed in adipose tissue, including adipocytes and macrophages, and plays an important role in the development of insulin resistance and atherosclerosis [3–5]. Chemical inhibition of FABP4 by a small molecule inhibitor could be a novel therapeutic strategy against insulin resistance, diabetes mellitus and atherosclerosis [6]. Recent studies have also demonstrated that neutralization of secreted FABP4 with an antibody to FABP4 could be a feasible approach for treatment of insulin resistance, type 2 diabetes mellitus, vascular injury and atherosclerosis [7–9].

FABP4 is secreted from adipocytes via a non-classical secretion pathway in association with lipolysis [7, 10] despite the lack of signal peptides [2] and acts as an adipokine for the development of insulin resistance, atherosclerosis and vascular remodeling [7, 8, 11]. Elevated serum concentration of FABP4 is associated with obesity, insulin resistance, hypertension, dyslipidemia, atherosclerosis, renal dysfunction, purine metabolism, heart failure and cardiovascular events [12–19]. Recent studies have also demonstrated modulation of FABP4 concentration by therapeutic drugs for hypertension, dyslipidemia and diabetes mellitus [20–26].

Dipeptidyl peptidase-4 (DPP-4) inhibitors, a class of antidiabetic drugs, have become standard drugs to improve hemoglobin A1c (HbA1c) levels in patients with type 2 diabetes mellitus [27]. It has been shown that DPP-4 inhibitors reduce intima media thickness, a marker of atherosclerosis [28, 29]. However, improvement of cardiovascular outcomes by adding DPP-4 inhibitors to usual care in patients with diabetes mellitus and cardiovascular diseases has not yet been proved [30–34]. DPP-4 inhibitors have distinct structures among the drugs [35]. Therefore, there might be an effect of each drug as well as a class effect of DPP-4 inhibitors. As a possible drug effect, anagliptin, a DPP-4 inhibitor, was reported to reduce low-density lipoprotein cholesterol (LDL-C) for 12–24 weeks regardless of the use of statins [36, 37]. Furthermore, treatment with anagliptin for 52 weeks was shown to be associated with a greater reduction in serum LDL-C levels compared to treatment with sitagliptin in patients with type 2 diabetes mellitus at high risk for cardiovascular events and with an LDL-C level > 100 mg/dL who were receiving statin treatment [38].

On the other hand, a monotherapy with sitagliptin and combination therapy with sitagliptin and a sulfonylurea agent for 12 weeks significantly decreased FABP4 concentration in patients with type 2 diabetes [23]. To reveal whether it is each drug effect or a drug class effect of DPP-4 inhibitors, we investigated the effects of treatment with the DPP-4 inhibitors anagliptin and sitagliptin for a relatively long period on FABP4 level in patients with type 2 diabetes mellitus at a high risk for cardiovascular events who were on statin therapy as a real-world setting.

## Methods

### Study patients

Study patients were retrieved from the Randomized Evaluation of Anagliptin vs. Sitagliptin On low-density lipoprotein cholesterol in diabetes (REASON) trial [38]. The detailed design including criteria for inclusion and exclusion in the REASON trial were previously reported [38, 39]. In brief, the trial was a multicenter, randomized, open-label, parallel-group design that investigated the effects of DPP-4 inhibitors, anagliptin (100 mg, twice daily) and sitagliptin (50 mg once daily), on reduction in LDL-C in adult patients with type 2 diabetes mellitus at high risk for cardiovascular events and with LDL-C levels of > 100 mg/dL who were receiving treatment with a statin. The recruited patients had previously documented atherosclerotic lesions in the coronary, intracranial, carotid, or other peripheral arteries. Randomization was centrally done with a web-based system using an algorithm balanced for sex, body mass index, hospitals, use of DPP-4 inhibitors prior to randomization, and levels of HbA1c and LDL-C. Antidiabetic drugs other than DPP-4 inhibitors at the beginning of the trial were concomitantly administered, and the original antidiabetic drugs were not replaced. During the period of the trial, antidiabetic and anti-dyslipidemia drugs were not added, and their dosages were not changed except for the dose of insulin.

The REASON trial was registered on Clinicaltrials.gov (NCT02330406) and was conducted in accordance with the Declaration of Helsinki and the Ethical Guidelines for Medical and Health Research Involving Human Subjects in Japan. The protocol and consent forms were approved by the institutional review boards at the University of the Ryukyus (No. 731) and each participating center. Written informed consent was obtained from all enrolled patients prior to randomization. In the first report of the REASON trial, anagliptin was reported to decrease LDL-C level to a greater extent than sitagliptin [38]. Sub-analysis studies using stored serum samples were planned in the protocol and were conducted according to the decision of the steering committee. The present study was one of the sub-analysis studies, and the effects of anagliptin and sitagliptin on FABP4 concentration were investigated. Among 313 patients who were enrolled in and completed the REASON trial, a total of 307 patients who were treated with anagliptin (n = 148; male/female: 89/59) or sitagliptin (n = 159; male/female: 93/66) for 52 weeks and for whom serum samples were stored were included in the present study.

### Measurements

Body mass index (BMI) calculated as body weight in kilograms divided by height in meters squared, waist circumference, past medical history, smoking status, alcohol consumption and use of concomitant drugs were evaluated at baseline. BMI was also checked at 52 weeks. Aspartate transaminase (AST), alanine aminotransferase, γ-glutamyl transpeptidase (γGTP), blood urea nitrogen, creatinine and fasting glucose were measured in each participating center at baseline and at 52 weeks. Estimated glomerular filtration rate (eGFR) was calculated from data for serum creatinine, age and sex using the following equation: eGFR (mL/min/1.73 m^2^) = 194 × serum creatinine^(−1.094)^ × age^(−0.287)^ × 0.739 (if female) [40]. Hemoglobin A1c (HbA1c) presented as the National Glycohemoglobin Standardization Program (NGSP) equivalent value, LDL-C measured by the direct method, total cholesterol, high-density lipoprotein cholesterol (HDL-C), triglycerides and insulin were analyzed at baseline and at 52 weeks in a core laboratory (SRL Inc., Tokyo, Japan). FABP4 concentration was measured using a commercially available enzyme-linked immunosorbent assay kit for FABP4 (BioVendor R&D, Czech Republic). The accuracy, precision and reproducibility of the kit have been described previously [12].

### Statistical analysis

Continuous variables were expressed as means with standard deviation (SD), means with standard error (SE) or medians with interquartile ranges. Categorical variables were expressed as the number with percentages and were compared between the treatment groups by the chi-squared test or Fisher’s exact test. Changes in parameters from baseline to 52 weeks in the groups were compared using the paired t-test, and differences in changes between the two treatment groups were analyzed by using the two-sample t-test. Correlations between two continuous variables were analyzed by using Pearson’s correlation coefficient. Multivariate linear regression models were constructed to explore independent parameters of change in FABP4 level. Age, sex, treatment group as well as variables with significant correlations determined by Pearson’s coefficient were incorporated in the models. The relationships were expressed with unstandardized regression coefficient, SE of regression coefficient and standardized regression coefficient (β). All statistical analyses were performed at an independent data center (Institute for Clinical Effectiveness, Kyoto, Japan) by study statisticians using JMP 13.1 (SAS Institute Inc, Cary, NC) and SAS 9.4 (SAS Institute Inc, Cary, NC). All P values were two-sided, and P < 0.05 was considered statistically significant.

## Results

### Characteristics of the patients at baseline

Baseline characteristics of the patients treated with anagliptin or sitagliptin are shown in Table 1. At baseline, 73–79% of the patients had hypertension and 45–46% of the patients had a previous history of coronary artery disease. A DPP-4 inhibitor was being administrated to 82% of the patients in the anagliptin group and 81% of the patients in the sitagliptin group prior to randomization. There was no significant difference between the anagliptin and sitagliptin treatment groups in the prevalence of habits of smoking and alcohol drinking, diagnosis including hypertension, coronary artery disease and stroke, and medications. There was also no significant difference in metabolic parameters except for BMI at baseline between the anagliptin and sitagliptin groups (Table 1).

**Table 1:** Background of the patients with type 2 diabetes mellitus (n = 307)

	Anagliptin	Sitagliptin	*P*
n (M/F)	148 (89/59)	159 (93/66)	0.77
Age (years)	68 ± 9	68 ± 9	0.91
Body mass index	26.7 ± 3.9	25.8 ± 3.6	0.04
Waist circumference (cm)	94.2 ± 11.1	92.7 ± 9.8	0.24
Smoking habit	71 (48)	92 (58)	0.08
Alcohol drinking habit	58 (39)	67 (42)	0.60
Diagnosis			
Hypertension	117 (79)	116 (73)	0.21
Coronary artery disease	68 (46)	71 (45)	0.82
Stroke	22 (15)	24 (15)	0.96
Medication			
Dipeptidyl peptidase-4 inhibitor^a^	122 (82)	128 (81)	0.66
Biguanide	74 (50)	71 (45)	0.35
Thiazolidinedione	22 (15)	29 (18)	0.43
α glucosidase inhibitor	19 (13)	27 (17)	0.31
Sulfonylurea	41 (28)	32 (20)	0.12
Glinide	7 (5)	2 (1)	0.09
Sodium–glucose cotransporter 2 inhibitor	25 (17)	16 (10)	0.08
Insulin	12 (8)	13 (8)	0.98
Statin	148 (100)	159 (100)	–
Strong statin^b^	118 (80)	121 (76)	0.44
Ezetimibe	16 (11)	11 (7)	0.23
Fibrate	9 (6)	7 (4)	0.51
Eicosapentaenoic acid	16 (11)	13 (8)	0.43
Angiotensin II receptor blocker	75 (51)	80 (50)	0.95
Angiotensin-converting enzyme inhibitor	18 (12)	10 (6)	0.07
Calcium channel blocker	72 (49)	68 (43)	0.30
β blocker	35 (24)	38 (24)	0.96
Diuretic	24 (16)	20 (13)	0.36
Mineralocorticoid receptor antagonist	6 (4)	6 (4)	0.90

Variables are expressed as number (%), mean ± SD or medians (interquartile ranges)

^a^The use before the study

^b^Indicates atorvastatin, rosuvastatin and pitavastatin

### Changes in metabolic parameters from baseline to 52 weeks

**Fig. 1:**
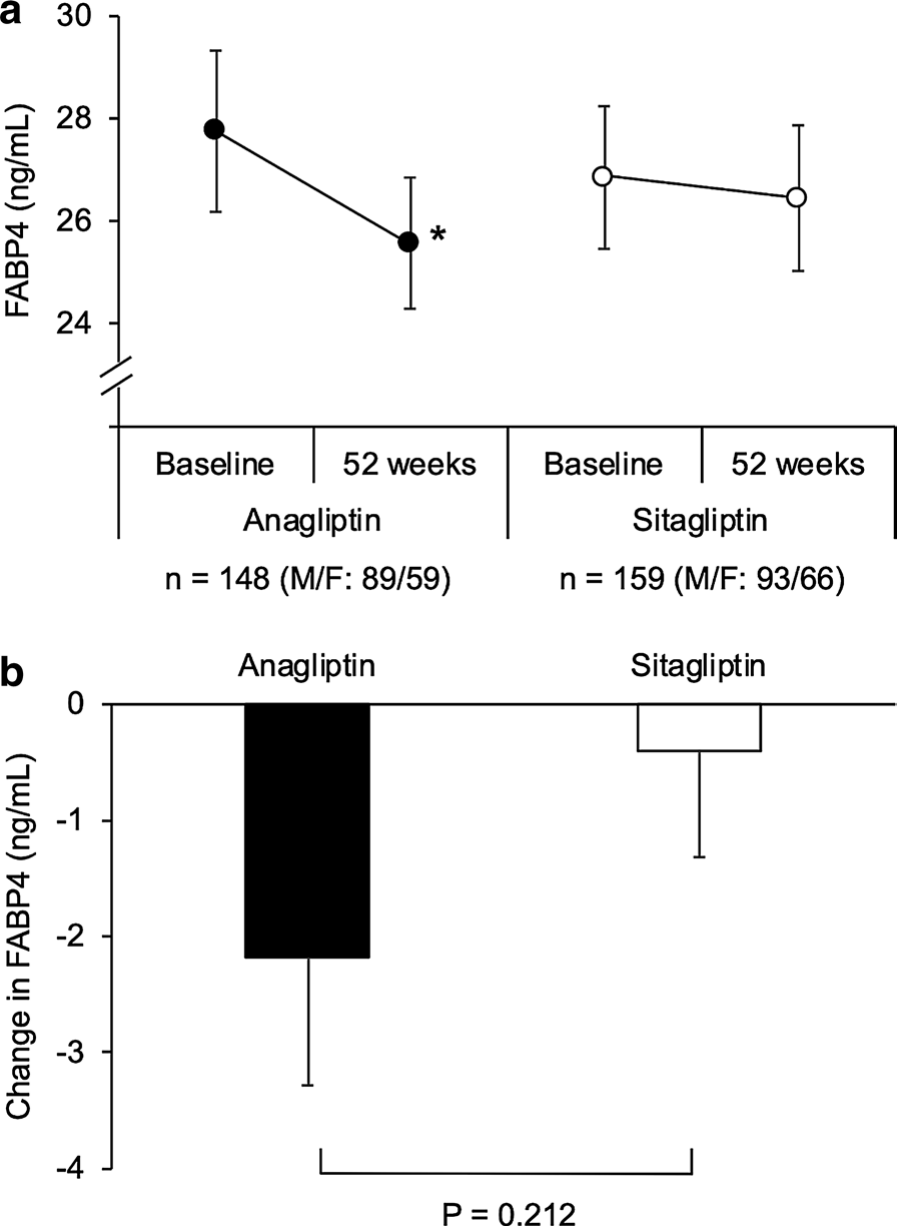
Effects of anagliptin and sitagliptin on FABP4 level. **a** Concentrations of FABP4 at baseline and 52 weeks in patients treated with anagliptin (n = 148, male/female: 89/59) and sitagliptin (n = 159, male/female: 93/66). **b** Comparison of change in FABP4 level between the anagliptin and sitagliptin treatment groups. Values are shown as mean ± SE. *P < 0.05

Treatment with anagliptin for 52 weeks significantly increased creatinine level and decreased BMI and levels of eGFR, total cholesterol and LDL-C (Table 2). On the other hand, treatment with sitagliptin for 52 weeks significantly increased AST, total cholesterol, HDL-C, fasting glucose and HbA1c. There were significant differences in changes in parameters including AST, total cholesterol, LDL-C and HDL-C from baseline to 52 weeks between the anagliptin and sitagliptin groups (Table 2). There was no significant difference in basal FABP4 levels in the anagliptin and sitagliptin groups, and serum FABP4 level was significantly decreased by 7.9% by treatment with anagliptin (27.8 ± 19.1 vs. 25.6 ± 15.5 ng/mL, mean ± SE, P = 0.049) and was not significantly decreased by treatment with sitagliptin (26.9 ± 15.5 vs. 26.5 ± 17.9 ng/mL, mean ± SE, P = 0.660) (Fig. 1a). No significant difference in change in FABP4 levels was found between the anagliptin and sitagliptin groups (− 2.1 ± 13.4 vs. − 0.4 ± 11.5 ng/mL, mean ± SE, P = 0.212) (Fig. 1b).

**Table 2:** Characteristics of the patients treated with sitagliptin or anagliptin for 52 weeks (n = 307)

	Anagliptin (n = 148)		*P*	Sitagliptin (n = 159)		*P*	*P*^a^
	Baseline	52 weeks		Baseline	52 weeks		
Body mass index	26.7 ± 3.9	26.5 ± 4.1	0.04	25.8 ± 3.6	25.8 ± 3.8	0.92	0.12
Waist circumference (cm)	94.2 ± 11.1	93.6 ± 10.9	0.30	92.7 ± 9.8	92.4 ± 9.6	0.56	0.69
AST (IU/L)	23 (18–30)	23 (18–29)	0.14	20 (18–25)	21 (18–27)	0.02	< 0.01
ALT (IU/L)	23 (17–34)	22 (14–33)	0.12	18 (15–26)	19 (14–26)	0.42	0.08
γGTP (IU/L)	30 (18–46)	27 (19–42)	0.16	24 (18–35)	24 (18–36)	0.12	0.07
Blood urea nitrogen (mg/dL)	16.7 ± 6.0	16.8 ± 5.3	0.89	17.1 ± 5.6	17.5 ± 5.5	0.30	0.51
Creatinine (mg/dL)	0.84 ± 0.27	0.86 ± 0.29	0.04	0.86 ± 0.29	0.87 ± 0.29	0.17	0.34
eGFR (mL/min/1.73 m^2^)	68.9 ± 20.7	66.8 ± 20.6	0.02	66.9 ± 18.5	65.7 ± 18.4	0.06	0.44
Total cholesterol (mg/dL)	190 ± 30	186 ± 28	0.02	186 ± 29	191 ± 27	0.01	< 0.01
LDL cholesterol (mg/dL)	112 ± 22	108 ± 22	0.01	110 ± 22	112 ± 21	0.15	< 0.01
HDL cholesterol (mg/dL)	54 ± 14	53 ± 13	0.43	55 ± 13	56 ± 13	< 0.01	0.01
Triglycerides (mg/dL)	136 (98–190)	134 (97–185)	0.80	114 (80–158)	110 (82–160)	0.22	0.55
Fasting glucose (mg/dL)	143 ± 42	147 ± 48	0.18	140 ± 39	146 ± 44	< 0.01	0.47
Insulin (µU/mL)	8.0 (5.7–12.0)	8.5 (5.3–13.3)	0.51	6.9 (4.5–11.0)	7.1 (4.7–11.5)	0.54	0.39
HbA1c (%)	7.1 ± 0.8	7.1 ± 0.9	0.71	6.9 ± 0.7	7.0 ± 0.9	0.04	0.28

Variables are expressed as mean ± SD or medians (interquartile ranges)

*AST* aspartate transaminase, *ALT* alanine transaminase, *eGFR* estimated glomerular filtration rate, *γGTP* γ-glutamyl transpeptidase

^a^For group difference in absolute change from baseline to 52 weeks

### Parameters associated with change in FABP4 level

**Fig. 2:**
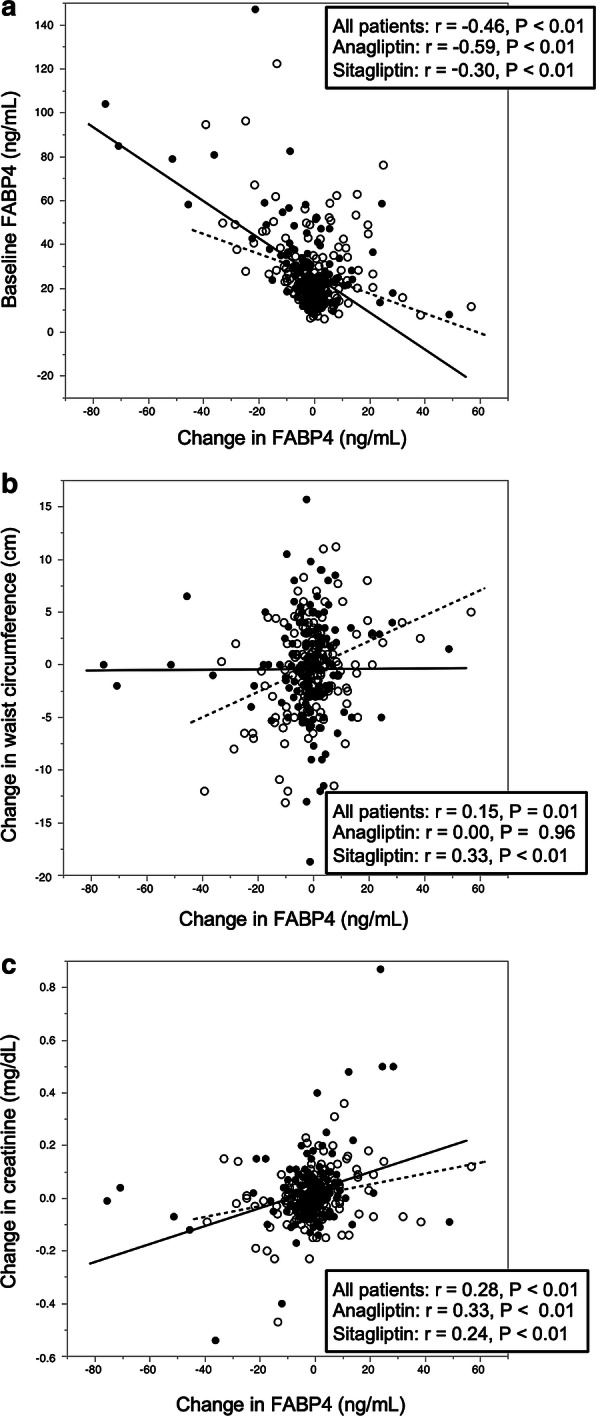
Correlations of change in FABP4 level with parameters. **a**–**c** Fatty acid-binding protein 4 (FABP4) level at baseline (**a**), change in waist circumference (**b**) and change in creatinine level (**c**) were plotted against change in FABP4 level in each subject (n = 307). Closed circles and solid regression line: anagliptin treatment group (n = 148), open circles and broken regression line: sitagliptin treatment group (n = 159)

As shown in Table 3, in all of the patients, change in FABP4 level was negatively correlated with FABP4 concentration at baseline (Fig. 2a) and changes in γGTP and eGFR and was positively correlated with changes in waist circumference (Fig. 2b), blood urea nitrogen and creatinine (Fig. 2c). No significant correlation of change in FABP4 with change in total cholesterol, LDL-C, HDL-C, triglycerides, fasting glucose, insulin or HbA1c was found (Table 3). Similar significant correlations of change in FABP4 level with the parameters except for change in waist circumference, that in γGTP and that in eGFR were found when the treatment groups were separately analyzed.

**Table 3:** Correlation analysis for ∆ FABP4

	Total (n = 307)		Anagliptin (n = 148)		Sitagliptin (n = 159)	
	r	P	r	P	r	P
Age at baseline	− 0.04	0.47	− 0.03	0.73	− 0.06	0.49
FABP4 at baseline	− 0.46	< 0.01	− 0.59	< 0.01	− 0.30	< 0.01
∆ body mass index	0.05	0.41	− 0.09	0.26	0.20	0.01
∆ waist circumference	0.15	0.01	0.00	0.96	0.33	< 0.01
∆ AST	− 0.10	0.07	− 0.02	0.83	− 0.21	0.01
∆ ALT	− 0.06	0.26	− 0.05	0.56	− 0.11	0.19
∆ γGTP	− 0.14	< 0.01	0.01	0.89	− 0.23	< 0.01
∆ blood urea nitrogen	0.21	< 0.01	0.20	0.01	0.21	0.01
∆ creatinine	0.28	< 0.01	0.33	< 0.01	0.24	< 0.01
∆ eGFR	− 0.18	< 0.01	− 0.20	0.01	− 0.15	0.06
∆ total cholesterol	0.04	0.47	0.02	0.80	0.03	0.66
∆ LDL cholesterol	0.05	0.42	0.00	0.97	0.08	0.35
∆ HDL cholesterol	− 0.05	0.41	− 0.15	0.07	0.04	0.59
∆ triglycerides	0.04	0.43	0.14	0.08	− 0.08	0.29
∆ fasting glucose	0.01	0.88	0.01	0.87	0.00	0.96
∆ insulin	0.05	0.40	0.00	0.97	0.15	0.06
∆ HbA1c	0.02	0.74	− 0.01	0.87	0.05	0.54

∆, change calculated as parameter in 52 weeks minus that in baseline

*AST* aspartate transaminase, *ALT* alanine transaminase, *eGFR* estimated glomerular filtration rate, *γGTP* γ-glutamyl transpeptidase

Multivariate linear regression models using age, sex, treatment group, FABP4 level at baseline and changes in waist circumference and creatinine as possible independent parameters showed that basal FABP4 level, change in waist circumference and change in creatinine were independent predictors of change in FABP4 level after adjustment of age, sex and treatment group (R^2^ = 0.294) (Table 4).

**Table 4:** Multivariate regression analysis for ∆ FABP4

	Regression coefficient	SE	Standardized regression coefficient (β)	*P*
Age	0.01	0.07	0.01	0.91
Sex (male)	− 1.82	1.30	− 0.07	0.16
DPP-4i (anagliptin)	− 1.81	1.23	− 0.07	0.14
FABP4 at baseline	− 0.31	0.03	− 0.45	< 0.01
∆ Waist circumference	0.29	0.14	0.10	0.04
∆ Creatinine	26.1	5.27	0.25	< 0.01

R^2^ = 0.294

∆, change calculated as parameter in 52 weeks minus that in baseline

*DPP-4i* dipeptidyl peptidase-4 inhibitor, *eGFR* estimated glomerular filtration rate, *FABP4* fatty acid-binding protein 4

## Discussion

### Main findings

The present study demonstrated that anagliptin, which has been reported to decrease LDL-C level [36–38], significantly reduced FABP4 concentration independent of change in HbA1c or LDL-C in patients with type 2 diabetes mellitus, dyslipidemia and existing atherosclerotic vascular lesions who were being prescribed statins. A statin was concomitantly used in all of the recruited patients, and angiotensin II receptor blockers and eicosapentaenoic acid were also administered in 50-51% and 8-11% of the patients, respectively. Those concomitant drugs have been shown to decrease FABP4 concentration [20–22]. It has been reported that treatment with sitagliptin alone and/or in combination with sulfonylurea decreases serum FABP4 level in patients with type 2 diabetes mellitus [23]. In the present study, more than 80% of the patients were pretreated with a DPP-4 inhibitor. The use of several kinds of pretreatment and concomitant drugs, which may modulate FABP4 concentration, is a possible reason for the lack of reduction in FABP4 level by treatment with sitagliptin in the present study. Reduction of FABP4 levels could be a class effect of DPP-4 inhibitors, though there had been no direct comparison of the effects of DPP-4 inhibitors on FABP4 levels. Anagliptin may be able to decrease serum FABP4 concentrations to a greater extent than sitagliptin in patients with type 2 diabetes mellitus and dyslipidemia who are on statin therapy.

### Possible mechanisms of reduction in FABP4 concentration by anagliptin

DPP-4 inhibitors have distinct structures among the drugs and include peptidomimetic and non-peptidomimetic agents [35], possibly leading to a drug class effect and each drug effect. Furthermore, DPP-4 inhibitors have been categorized into three classes of binding pocket on the basis of their binding subsites [41, 42]. The selectivity, specificity and extent of action of each drug may cause specific and pleiotropic effects. However, little is known about the differences in those effects among different types of DPP-4 inhibitors.

A possible drug effect of anagliptin is the reduction of LDL-C [36–38]. Experimental studies showed that anagliptin reduced cholesterol synthesis due to downregulation by sterol regulatory element-binding protein 2 in the liver [43] and inhibited absorption of cholesterol in the small intestine [44], leading to the reduction of LDL-C level. In a human study using a small number of drug-naïve patients with type 2 diabetes and LDL-C > 120 mg/dL (n = 30), anagliptin significantly decreased a cholesterol synthesis marker, lathosterol, without affecting cholesterol absorption markers including campesterol, sitosterol and cholestanol [45]. Furthermore, a previous sub-analysis using data from the REASON trial demonstrated that anagliptin reduced LDL-C level by suppressing excess cholesterol synthesis even when it was used in combination with statin therapy [46]. Taken together, these findings suggest that anagliptin exerts a hepato-protective effect beyond its glycemic-lowering action. However, no association of change in FABP4 level with change in LDL-C level was found in the present study. A distinct mechanism by which FABP4 level is decreased by anagliptin needs to be addressed in the future.

### Possible drug class effect of DPP-4 inhibitors on FABP4 concentration

A previous study demonstrated that the mechanism of the decrease in FABP4 level by a DPP-4 inhibitor is, at least in part, via reduction in the expression and consecutive secretion of FABP4 in adipocytes by direct inhibition of soluble DPP-4 as an adipokine [23]. It has also been reported that postprandial hyperglycemia and fluctuation of glucose level, which may cause sympathetic nerve activation [47], are improved by DPP-4 inhibitors [48]. Since FABP4 is secreted from adipocytes in association with lipolysis [7, 10], DPP4 inhibitors may decrease FABP4 concentration by inhibiting FABP4 secretion from adipocytes in association with sympathetic tone-mediated lipolysis. However, glucose fluctuation assessed by 1,5-anhydroglucitol level and/or continuous glucose monitoring was not investigated in the present study. DPP4 inhibitors have also been shown to suppress inflammatory responses through activation of NACHT, LRR and PYD domains-containing protein 3 (NLRP3) inflammasome and c-jun N-terminal kinase [49, 50] and to decrease levels of tumor necrosis factor and interleukin 6 [51, 52], which are known to increase lipolysis in adipocytes [53]. This could be another indirect mechanism of the decrease in FABP4, though those factors were not evaluated in the present study.

### Change in FABP4 level

In all of the patients, change in FABP4 level was independently associated with basal FABP4 level and changes in waist circumference and creatinine after adjustment of age, sex and the treatment group (Table 4). There was no significant difference between basal FABP4 levels in the anagliptin and sitagliptin treatment groups. It has been reported that FABP4 level is independently associated with adiposity [12, 13] and a decline of renal function [13, 17]. In the present study, treatment with anagliptin, but not treatment with sitagliptin, significantly decreased BMI as an index of adiposity even in concomitant with a significant increase in creatine level, suggesting that reduction of adiposity by anagliptin, but not sitagliptin, leads to a decrease in FABP4 level.

### Limitations

First, no washout of DPP4 inhibitors before the beginning of trial was performed. Pretreatment with DPP-4 inhibitors may have affected the FABP4 concentration. Second, most of the study subjects were being treated at baseline with several drugs including antidiabetic drugs [23–26], statins [20], angiotensin II receptor blockers [21] and omega-3 fatty acid ethyl esters [22], which have been reported to affect circulating FABP4 concentration. Therefore, such drugs might have modulated the change in FABP4 level. However, randomized allocation should balance these drugs between groups and minimize the effects. Third, the present study lacked a placebo control group. Interventional studies using larger number of subjects and a placebo-control design are necessary for determining the impact of DPP-4 inhibitor treatment on circulating FABP4 level and the relationship between change in FABP4 level and clinical benefit of DPP-4 inhibitors. Lastly, the impact of a 7.9% reduction of FABP4 level by anagliptin is unclear, though previous studies using experimental models showed metabolic and cardiovascular effects of circulating FABP4 and neutralizing antibody of FABP4 [7, 8, 11, 54].

## Conclusion

Anagliptin decreases FABP4 concentration independent of change in hemoglobin A1c or LDL-C in patients with type 2 diabetes mellitus at a high risk for cardiovascular events who are on statin therapy. This study provides additional evidence for the importance of careful selection of the drug regimen for patients with diabetes mellitus and other risk factors in order to prevent cardiovascular events. Reduction of FABP4 level as a possible class and pleiotropic effect of DPP-4 inhibitors might be beneficial for patients with metabolic and cardiovascular diseases. A further understanding of the mechanisms by which FABP4 level is changed by pharmacological modulation may enable the development of new therapeutic strategies for cardiovascular and metabolic diseases.

## Data Availability

The datasets analyzed during the current study are available from the corresponding author on reasonable request (furuhasi@sapmed.ac.jp).

## Abbreviations

AST: Aspartate transaminase
BMI: Body mass index
DPP-4: Dipeptidyl peptidase 4
eGFR: Estimated glomerular filtration rate
FABP: Fatty acid-binding protein
γGTP: γ-glutamyl transpeptidase
HbA1c: Hemoglobin A1c
HDL-C: High-density lipoprotein cholesterol
LDL-C: Low-density lipoprotein cholesterol
SD: Standard deviation
SE: Standard error
